# A New Approach to Antivenom Preparation Using Chitosan Nanoparticles Containing *EchisCarinatus* Venom as A Novel Antigen Delivery System

**Published:** 2017

**Authors:** Farya Mirzaei, Naser Mohammadpour Dounighi, Mohammad Reza Avadi, Mehdi Rezayat

**Affiliations:** a *Department of Nanotechnology, Faculty of Science, Pharmaceutical Sciences Branch, Islamic Azad University, Tehran, Iran. *; b *Department of Human Vaccines and Serum, Razi Vaccine and Serum Research Institute, Agricultural Research, Education and Extension Organization, Karaj, Iran. *; c *Department of Research and Development, Hakim Pharmaceutical Company, Tehran, Iran. *; d *Department of Nanotechnology, Tehran University of Medical Science, Tehran, Iran.*

**Keywords:** Chitosan, Nanoparticles, *Echiscarinatus* venom, Adjuvant, Hyperimmune plasma

## Abstract

In recent years, use of biodegradable polymers based nanoparticles has received high interest in the development of vaccines delivery vehicles. The aim of study was to prepare chitosan nanoparticles (CS NPs) for loading *Echis carinatus * (EC) venom and evaluate their potential as an adjuvant and antigen delivery system on a pilot scale. CS NPs were prepared using ionic gelation method, and their characteristics were optimized. Venom-loaded CS NPs prepared under optimum conditions and traditional venom-loaded adjuvants were used to hyperimmunization of horse. Under optimum conditions, particle size, polydispersity index (PDI), and zeta potential of CS NPs were 127.9 ± 15 nm, 0.29, and +19.8 ± 1.92 mV, while those of venom-loaded CS NPs were 182.4 ± 20 nm, 0.35, +26.8 ± 1.98 mv, respectively. All CS NPs had integrated surface and good morphology. Optimum loading concentration of EC venom was 500 µg/mL. The loading capacity (LC) and loading efficiency (LE) were 87% and 94%, respectively, and release profile of venom-loaded CS NPs showed suitable correlation with Higuchi kinetics. Stability test showed good stability of the venom encapsulated in CS NPs. Furthermore, antivenom plasma obtained using the new antigen delivery system had significantly higher potency (*P *< 0.05) for neutralizing the venom than that obtained using conventional system. These results suggested that venom-loaded CS NPs could be a suitable alternative to conventional adjuvant for development antivenom.

## Introduction

CS NPs have different desirable properties compared with conventional adjuvants and antigen delivery systems (w/o emulsion). These include excellent versatility in preparing derivatives with different materials and loading ingredients; lower cost; easy access to polymers; high surface to volume ratio because of its small particle size in same volume, which results in high loading capacity and long-term release (a crucial advantage for manufacturing vaccines and drugs); improved stability of entrapped agents; flexible modes of administration; and appropriate adjuvant properties ideal for generating antigen delivery system. On the other hand, antigen-loaded CS NPs reduce the number of injections required, have fewer side effects (such as pain and ulcer), deliver large concentrations of intact antigens into the body, and allow targeted delivery of antigens to immune cells, thus stimulating the immune system. Therefore, they can be use as good alternatives to conventional systems for manufacturing vaccines (2, 7 and 16-19).

In this study, we prepared CS NPs containing *Echis carinatus* (EC) venom, one of the most poisonous and dangerous snake venoms that has fast and powerful effect on the blood system(20-24),as a novel antigen delivery system for use in the hyperimmunization step of antivenom manufacturing process. Hyperimmune plasmas were obtained by hyperimmunizing horses with the new nanoantigen delivery system and conventional adjuvant, and venom neutralization potencies of the two hyperimmune plasmas were measured and compared.

## Experimental

*Chemical and biological materials*

In this study, we used CS (Sigma-Aldrich, USA) having medium molecular weight (MW) of 389.356 KDa, deacetylation degree (DD) of 75%-85%, and viscosity of 1326.03 cp; 95% acetic acid (Merck, Germany), and TPP (Sigma-Aldrich, USA). EC venom, in the form of freeze-dried powder, and EC venom entrapped common w/o emulsion adjuvant was obtained from the Razi vaccine and sera research institute (Alborz, Iran). All other chemicals were analaytical grade. 

*Animals*

Two types of animals were used in different stages of the research. Saurian mice with weight 18-20 g were used in toxicity test and evaluation of neutralization potency of antivenom. Horses (weight, 300-500 kg; age, 4-6 y) were used to prepare hyperimmune plasma. In all, four horses were used (two for the conventional method and two for the novel nanoantigen delivery system). The study was reviewed by the Razi Vaccine and Serum Research Institute (Karaj, Iran), and all animals were handled in accordance with institutional guidelines.

*Toxicity test*

The toxicity of EC venom was determined using Finney method, and lethal dose 50% (LD_50_) was determined using Spearman-Karber analytical calculations (25). Brifely, for toxicity test, mice were divided into 6 groups, containing four mice in each group. Different amounts of venom were injected intravenously into in each group of mice. The numbers of death of mice in each group was recorded after 24 h and the lethality value (LD_50_) of venom calculated.

*Preparation of CS NPs*

CS NPs were prepared using the procedure reported by Calvo* et al.* , based on the gelation of CS with TPP anions (12). Different concentrations of CS (2, 2.5, and 3 mg/mL) were dissolved in an aqueous solution of acetic acid. The concentration of acetic acid in the aqueous solution was 1.5 times higher than each concentration of CS. Then, aqueous solution of CS was added drop wise into TPP solution (1 mg/mL) with constant stirring and at room temperature. This resulted in the formation of an opalescent suspension. Finally, CS NPs were separated by centrifugation at 61860 × g and 5 ± 2 °C for 20 min. After centrifugation, CS NPs were freeze dried (temperature of −50 °C and a pressure of 0.08 mbar), and stored at 4-8 °C until further analysis. EC venom-loaded CS NPs were prepared by adding CS solution to TPP solution containing various concentrations of EC venom. In this study, effects of various factors such as polymer and venom concentrations, polymer and cross-linker solutions volume ratio, homogenizer speed, temperature, pH, and droplet addition flow rate on the characteristics of CS NPs were investigated while establishing the optimum conditions for preparing CS NPs. Only a single factor was altered at a time while others were kept constant. Additional parameters of CS NPs, such as morphology, structure, encapsulation, and loading efficiency (LE), were also studied.

*Morphology, size, and surface charge characterization*

Morphology, surface characteristics, and size of CS NPs were examined using scanning electron microscopy (SEM-633, SEMTech, USA). The particle size, size distribution and zeta potential of nanoparticles were measured using a Zetasizer (Malvern Instruments, UK). The particle size and zeta potential were determined based on the dynamic light scattering technique (DLS) and electrophoretic mobility of CS NPs in aqueous suspensions, respectively.

*FTIR measurements*

Structures of CS, venom, CS NPs, and venom-loaded CS NPs were evaluated using Fourier transform infrared (FTIR) spectroscopy. IR spectra of samples were determined using KBr pellets and recorded using FTIR spectrophotometer (FTIR- 410, Jasco, Colchester, United Kingdom).

*Loading efficiency (LE) and loading capacity (LC) of EC venom*

LE and LC of EC venom in venom entrapped CS NPs were measured indirectly by determining free venom in the supernatant. For this, CS NP suspension was centrifuged at 61,860 × g and 12 ± 2 °C for 30 min. Protein concentration in the supernatant was estimated using Bradford protein assay (26). LE and LC values were calculated using the following equations (27):

LC = (A − B)/C × 100                                        (1)

LE = (A − B)/A × 100                                         (2)

Where A is the total amount of venom, B is the free venom, and C is NP weight.

*In-vitro release studies*

The release profile of EC venom from CS NPs and common w/o emulsion adjuvant system was determined as follows: Venom-loaded CS NPs and venom-entrapped common adjuvant were divided into several test tubes (1 mg CS NPs and 1 mL common system in each tube). Next, one mL phosphate-buffered saline (PBS) 0.2 molar pH 7.4 was added to each test tube. The tubes were shaker incubated at 200 rpm and 37 ± 1 °C. Samples were removed at scheduled time intervals (until 194 h), nanoparticle samples centrifuged at 61860 × g and 5 ± 2 °C for 20 min and common system samples filtered by 0.2 µm membrane filter (Minisart^®^, Sartorius, Germany). Protein concentration in the supernatant and filtrate was estimated using the Bradford method.

*Stability study of venom-loaded CS NPs*

Prior to acceptance and approval of any pharmaceutical product, it is important to maintain product quality, safety, and efficacy throughout the product shelf life. Accelerated stability test (storage conditions, 25 ± 2 °C; relative humidity, 60 ± 5%) was performed to investigate the stability of venom-loaded CS NPs (28). In carrying this out,samples of venom-loaded CS NPs were stored in the aforementioned special conditions and studied for 0, 2, 4, and 6 months, respectively. Biological stability of the venom encapsulated in CS NPs was determined by evaluating the toxicity of the venom released from the NPs during incubation (25). Physicochemical stability of venom-loaded CS NPs was determined by evaluating zeta potential, particle size, and particle size distribution by using Malvern zeta-potential analyzer. Morphology and surface characteristics of venom-loaded CS NPs were studied using scanning electron microscopy (SEM).

*Hyperimmunization of animals*

Hyperimmunization was performed, in two steps each for 56 days, with one week injection intervals and one month rest period between hyperimmunization steps, using both new nanoantigen delivery system and the conventional method. This was followed by bleeding of the animals in order to obtain the hyperimmune plasma (29).

*Potency test of the hyperimmune plasma*

The mice were given an intravenous injection of incubated venom-plasma mixture inorder to measure the neutralization potency of lethality. Mixtures containing varying dilutions of the venom (2.6, 3.2, 4, 5, 6.2, 7.8, 9.8 and 12.2LD_50_/mL) and constant concentrations (1:3 of mixture volume) of hyperimmune plasma were prepared in normal saline and incubated at 37°C for 1 h. Aliquots (0.5 mL) of mixtures were injected intravenously into mice. Deaths were recorded over 96 h, and the potency of hyperimmune plasma was estimated. The potency of antivenins under test was expressed in terms of LD_50_. The weight in mg equivalent to the LD_50_ of the venom should be neutralized by a specific quantity of the plasma based on the protection of a stated proportion of animals (*e.g.*, 100%). Serum potency was expressed as the largest amount of venom neutralized by 1 mL of serum (29). Venom neutralization potency of hyperimmune plasmas prepared by both methods was measured and compared.

*Statistical analysis*

In this study all experiments were analyzed at least in triplicate. Data are expressed as mean ± SD. Statistical analysis was carried out using Student’s *t*-test.

## Results and Discussion

*Toxicity of the venom*

The toxicity (LD_50_) of crude EC venom was50 μg/mice. This result showed a high toxicity for the EC venom. In the other hand, the mortality rate* of Echiscarinatus* envenomation is 10-20 %, if there is no immediate effective treatment (30). Therefore, it is necessary to develop an advanced antigen delivery system for the preparation of effective antivenom against the venomous snake specie *Echiscarinatus*. 

*Physicochemical characterization of CS NPs*

**Figure 1:**
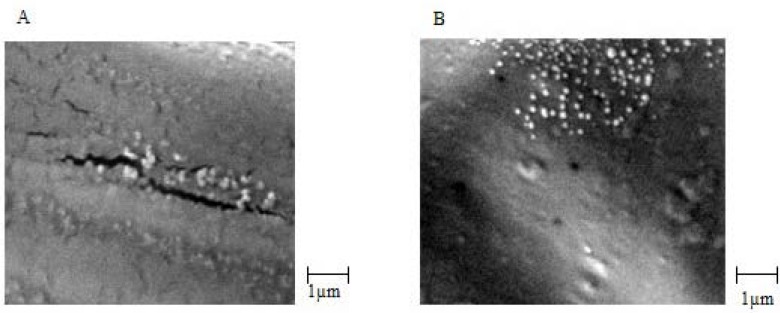
SEM images: (A) chitosan nanoparticles (CS 2 mg/mL, TPP 1 mg/mL) and (B) EC venom loaded chitosan nanoparticles (CS 2 mg/mL, TPP 1 mg/mL, ECV 500 µg/mL

CS NPs were prepared by ionic cross-linking (ionic gelation) of oppositely charged CS and TPP negative ions. Particle size, particle size distribution, and zeta potential of CS NPs were evaluated using the Zetasizer. In this study, suitable characteristics of CS NPs were achieved by polymer concentration of 2 mg/mL, cross-linker of 1 mg/mL, and stirring rate of 1200 rpm. The particle size, Polydispersity Index (PDI), and zeta potential of CS NPs and venom-loaded CS NPs prepared under optimum conditions showed in Table 1. SEM images showed that CS NPs had integrated surface and good morphology. These particles were approximately spherical, with an almost homogeneous structure. In addition, SEM images showed that the mean particle size of CS NPs was 110 ± 10 nm and that of venom-loaded CS NPs was 215 ± 20 nm, which were approximately similar to the results of Zetasizer (Figure 1). It is noteworthy here that the size of nanoparticles increased with association of venom molecules due to the presence of large size molecules in venom composition. These results are in line with previous reports (8). The results showed that entrapment of venom only slightly enhanced zeta potential of nanoparticles. This could be attributed to the increase of particles size and surface area and its consequences are similar to those previously reported by Huang *et al. *(31).

**Figure 2:**
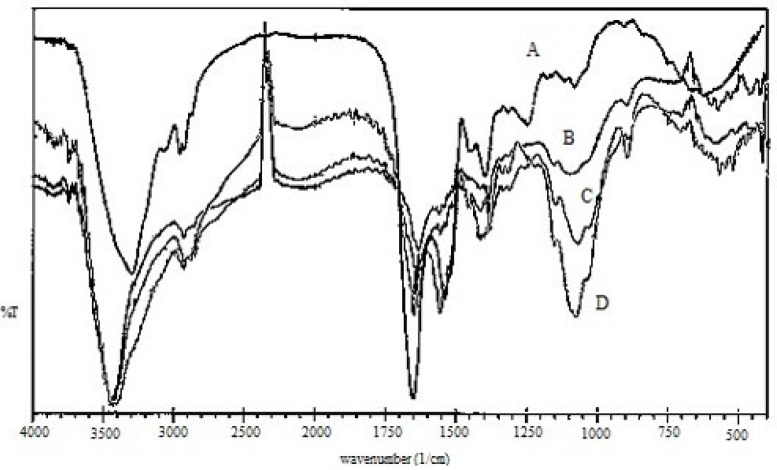
FTIR spectrums: (A) EC venom, (B) chitosan, (C) chitosan NPs (CS 2mg/mL, TPP 1 mg/mL) and (D) EC venom-loaded CS NPs (CS 2 mg/mL, TPP 1 mg/mL, ECV 500 µg/mL

Figure 2 illustrates the Fourier transform-infrared (FTIR) spectra of (A) EC venom, (B) CS, (C) CS NPs, and (D) venom-entrapped CS NPs. Three characteristic absorption bands observed for CS (Figure 2B) at 3413, 1630, and 1382 cm^−1^ were due to -NH, amide I, and amide III groups, respectively, present in CS (32). In the CS spectra, the strong and broad band in the 3200-3450 cm^−1^ range corresponded to amine and hydroxyl groups (-OH stretching and intermolecular hydrogen bonding). The peak near 2930 cm^−1^ was caused by -OH stretching. The intense peak at 1382 cm^−1^ was caused by -NH stretching of amide in the fingerprint region of the spectra; symmetric stretching of C-O-C was observed around 1070 cm^−1^. Absorption band for carbonyl (C=O) stretching of the secondary amide was observed near 1658 cm^−1^. The peak at 570 cm^−1^ was caused by the saccharide structure of CS.

The peak at 3413 cm^−1^ corresponded to the asymmetric and symmetric stretching vibrations of N-H in pure CS. This peak shifted to lower wave numbers at 3400 cm^−1^ and was broader and stronger for CS NPs (Figures 2C and 2D), indicating hydrogen bonding between these groups and TPP. The peaks for CS and CS NPs were broader in this region because of the contribution of -OH stretching peaks and hydrogen bonding (33). For CS NPs, the peak at 1630 cm^−1^ caused by -NH_2_bending vibration shifted to 1645 cm^−1^. Knaul observed a similar result for CS film treated with NaH_2_PO_4_ and attributed it to the linkage between phosphoric and ammonium ions (34). Therefore, it was assumed that tripolyphosphoric groups in TPP were linked to ammonium group in CS, resulting in an enhancement of the inter and intra-molecular actions in CS NPs. 

Characteristic peaks of FTIR spectra of EC venom showed peaks around 1650, 1541and 3305 cm^−1^, reflecting the acetylamino I, acetylamino II and NH_2_ groups, respectively (35). Acetylamino I at 1650 cm^−1^ and acetylamino II at 1541 cm^−1^ of EC venom (Figure 2A) overlapped amide I at 1649 and 1558 cm^−1^ of CS NPs; hence, intensive peaks appeared for venom-loaded CS NPs (Figure 2D).

FTIR spectra of venom-entrapped CS NPs (Figure 2D) demonstrated that stretching vibrations of -OH and -NH_2_ at 3400 cm^−1^ were broader. The intense peak at 1414 cm^−1^ belonged to C-N stretching. For venom-loaded CS NPs, the 1650 cm^−1^ peak of acetylamino shifted to 1658 cm^−1^ perhaps because of the cross-linking between EC venom and CS (34, 36).

**Table 1:** Physicochemical characteristics of CS NPs and venom loaded CS NPs prepared under optimum conditions, mean ± SD, n = 3 (CS 2 mg/mL, TPP 1 mg/mL, ECV 500 µg/mL

**NPs**	**Mean diameter, nm (n=3)**	**PDI**	**Zeta potential (mV)**
CS NPs	127.9 ± 15	0.29	+19.8 ± 1.92
Venom loaded CS NPs	182.4 ± 20	0.35	+26.8 ± 1.98

**Table 2.** Results of accelerated stability study of venom loaded CS NPs (CS 2 mg/mL, TPP 1 mg/mL, ECV 500 µg/mL), Storage conditions, 25 ± 2 ˚C/60 ± 5% RH*.

**Incubation ** **time** **months**	**Physicochemical characteristics**			**Biological activity**
	**Zeta potential** **(mV)**	**Size** **(nm)**	**Morphology**	**LD**_50_ **(µg/mice)**
zero time	+26.8 ± 1.98	182.4 ± 20	Spherical/integral surface normal distribution	50
2 months	+26.8 ± 1.88	182.4 ± 20	Spherical/integral surface normal distribution	50
4 months	+20.2 ± 2.11	184 ± 40	Spherical/integral surface acceptable size distribution	50
6 months	+20.2 ± 2.22	240 ± 80	Semi spherical/integral surface slight aggregation	50

* Relative humidity.

**Table 3:** Results of venom lethality neutralization potency test of antivenom plasma after first and second hyper-immunization by novel and common methods, mean ± SD, n = 3.

**Hyperimmunization** **steps**	**First hyperimmune plasma**		**Second hyperimmune plasma**	
	**Common method**	**Novel method**	**Common method**	**Novel method**
Potency (mice LD_50_/mL)	12 ± 2.5	12 ± 3.0	20 ± 2.0	36 ± 2.0

**Figure 3:**
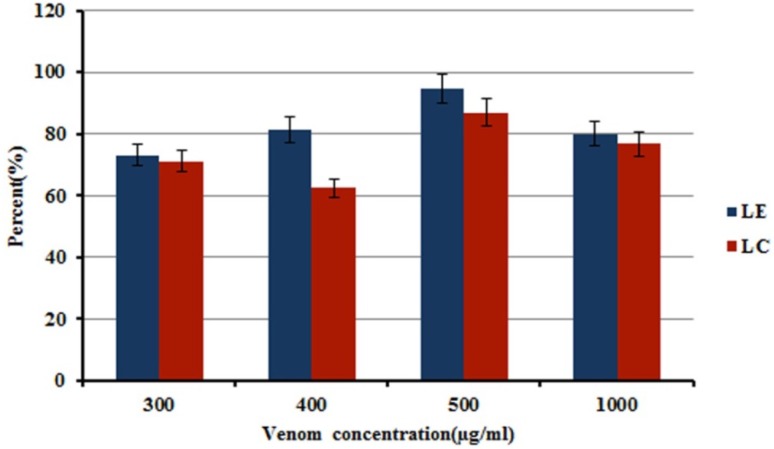
Effect of initial EC venom concentrations on loading efficiency and capacity (CS 2 mg/mL, TPP 1 mg/mL)

**Figure 4:**
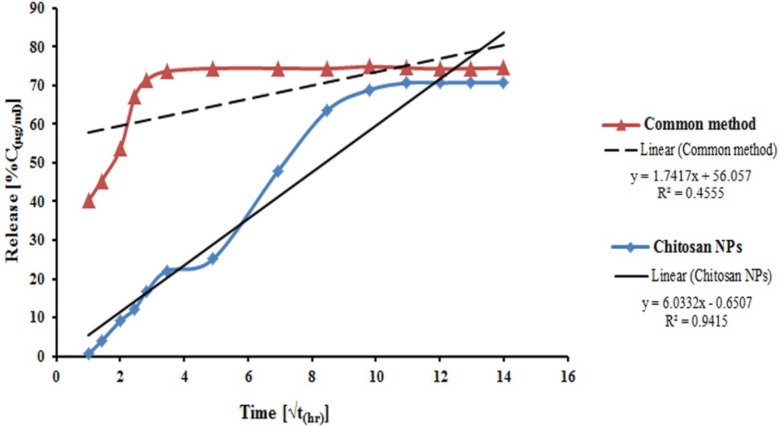
*In-vitro* release profile of EC venom according to Higuchi’s release kinetic formula from venom loaded NPs (CS 2 mg/mL, TPP 1 mg/mL, ECV 500 µg/mL, LC 87%) and venom-entrapped common system

**Figure 5.**
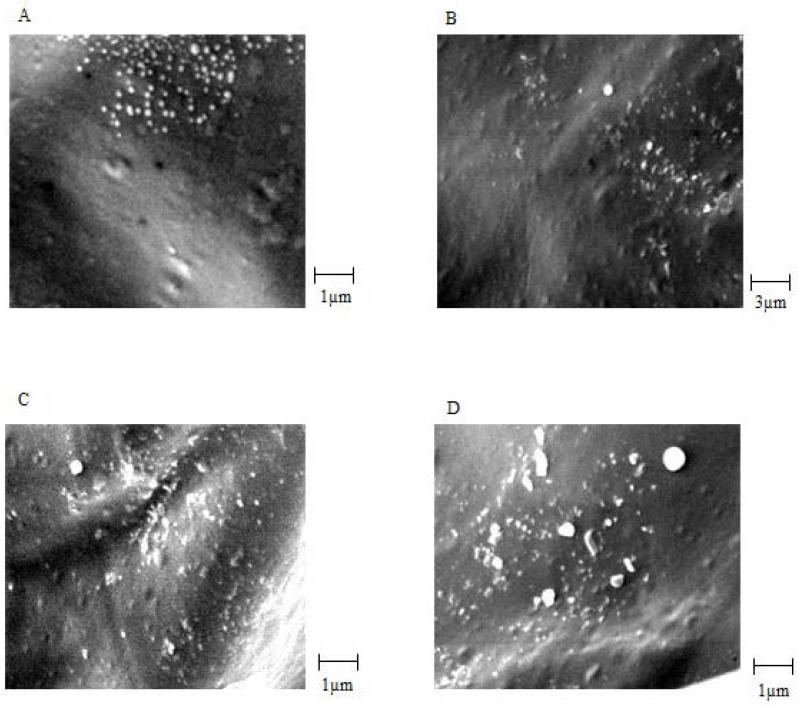
SEM images of venom-loaded CS NPs in stability test: (A) At 0 time, (B) after 2 months, (C) after 4 months and (D) after 6 months

*Evaluation of Loading Capacity (LC) and Loading Efficiency (LE)*

As illustrated in Figure 3, LC and LE increased with an increase in the initial venom concentration (from 300 to 500 μg/mL). However, LC and LE decreased when venom concentration increased to 1000 μg/mL. It might be that enhancing initial concentration of the venom until 500 μg/mL leads to improvement of LE and LC, for the reasons of both chemical interaction and physical entrapment induction. However, higher concentrations of venom by increase of viscosity of medium, relative saturation of chemical interaction sites, coupled with physical limitation for entrapment spaces resulted in a decrease of LE and LC. It could be understood that, increase in viscosity of medium made the encapsulation of venom more difficult because the enhanced viscosity might result in a reduced diffusion rate of venom in the chitosan solution. The same explanation was used to discuss the decrease in LE of dexamethasone (37), LE and LC of BSA (38) and lysozyme (39) in CS NPs by higher concentrations of the mentioned agents.

Owing to a suitable LC and LE, an initial concentration of 500 μg/mLwas selected as the optimum venom concentration for preparing venom-entrapped CS NPs. The LE and LC, with this initial venom concentration were 94 and 87%, respectively (Figure 3).

*In-vitro release profile of venom-loaded CS NPs and traditional system*

*In-vitro* release profile of venom-entrapped CS NPs was evaluated using NPs, prepared under optimum conditions (2 mg/mL CS , 1 mg/mL TPP and 500 μg/mL venom initial concentration), with an LC of 87%, and PBS pH of 7.4 as a release medium (40). The release patterns of venom-entrapped CS NPs and traditional emulsion system are shown in Figure 4. The venom-entrapped CS NPs demonstrated an initial slow release of the venom in the first 16 h and then a relatively constant release rate until 168 h. Ultimately, approximately 70% of the venom was released from venom-loaded CS NPs within 8 days. As illustrated in Figure 4, the common venom-loaded adjuvant system indicated a high burst release of about 40% of entrapped venom at the zero time, and 71% of venom was released rapidly for 8 h. From the results of release profile of venom from CS NPs, it is speculated that EC venom is mainly entrapped in NPs and only a small amount of venom is adsorbed on the surface of NPs (41). 

The results demonstrated a suitably sustained release profile for venom from venom-loaded CS NPs prepared in this study and very fast release from emulsion system. The rapid release of venom from common emulsion system could result to high concentration of venom in the injection site and consequently, serious local lesions. Conversely, the immune system will be in contact with the venom for a short period, because of rapid release and degradation of the released venom by enzymes of body fluids. One important situation in vaccine delivery systems from the point of immunogenicity is a sustained release of antigen from NPs. Therefore, this system can serve as a potential and useful effective adjuvant in the preparation of an anti-venom. 

Venom release profiles from venom-loaded CS NPs were assessed by different release kinetics: zero order, first order, Higuchi model, Korsmeyer-Peppas model, and Hixson-Crowell model. Obtained data showed that this is closest to Higuchi model. Higuchi in 1961 put forth the first mathematical model to describe drug release from a matrix system. Although the model was initially conceived for planar systems, it was later extended to different geometrics and porous systems (42).

Release profile of venom-loaded CS NPs (Figure 4) indicated that CS NPs with a huge large surface area could adsorb EC venom, thus allowing the venom to be released easily in the first few hours and at a constant rate later because of the slow degradation of CS NPs, and consequently the release of entrapped venom (11). 

*Accelerated stability studies*

Biological stability of EC venom encapsulated in CS NPs was measured using the Finney method based on a method used for venom before loading. The biological activity of the venom was expressed in terms of LD_50 _(25). For physicochemical stability, average particle size, surface potential, and morphology were measured. The results are summarized in Table 2.SEM images of venom-loaded CS NPs in stability test at zero time, after 2, 3, 4 and 6 months are shown in Figures 5A, 5B, 5C and 5D, respectively. The results of the accelerated stability studies showed that venom-entrapped CS NPs had a highly favourable biological and acceptable physicochemical stability for 6 months. After 6 months, the zeta potential of venom-loaded CS NPs decreased slightly (Table 2), resulting in a slight aggregation of venom-loaded CS NPs (Figure 5D). However, the biological activity of the venom released from CS NPs after 6 months was similar to zero time and the venom remained intact during accelerated stability study as shown in Table 2.

The results of this study revealed good physicochemical and biological stability of venom loaded NPs (43). At this point , it is important to notice the school of thought of the effect of freeze drying on enhancing stability of venom entrapped NPs (44).

*Lethality neutralization potency of hyperimmune plasma*

In this study, both the hyperimmunization procedures showed the same lethality neutralization resulted in the first stage of hyperimmunization. After the second stage of hyperimmunization of animals, by using a similar dose of antigen in both methods, the novel system showed higher potency (~ 2 fold) for neutralizing the venom than the conventional system (Table 3). The mean venom lethality neutralizing titer in the novel system group was significantly greater than the traditional adjuvant groups (*P *< 0.05).

These results indicated that in addition to having favourable features such as biodegradability, biocompatibility, ease of preparation, good stability, and long-term preservation of antigens without changes in their biological and physiological properties, the novel antigen delivery system could elicit a good immune response and produce hyperimmune plasma, having high potency to neutralize the venom compared with that obtained using conventional adjuvants. Moreover, the conventional antigen delivery system with W/O emulsion has some setbacks, such as instability, complex preparation process, time consuming, expensive, adverse reactions at injection site (such as scars and pain) in animals, and damage to loaded antigens (3, 45-47).

## Conclusions

EC venom can be successfully loaded into CS NPs, with high LC and LE. CS NPs and venom-loaded CS NPs prepared in this study had the desired characteristics. In addition, the release profile of the encapsulated venom was sustained and had kinetics closest to the Higuchi model, an appropriate model for sustained release systems. Accelerated stability study of venom-loaded CS NPs showed a good ability of this system to preserve the activity of the antigen up to 6 months. The results of hyperimmunization of animals on a pilot scale showed that the novel venom-loaded CS NPs significantly stimulated the immune system compared with conventional adjuvants. Antivenom plasma prepared using the new antigen delivery method had higher potency (~ 2 fold) for neutralizing the venom than that prepared using the conventional method. Thus, it can be suggested that venom-loaded CS NPs can be used as an alternative to conventional adjuvants for manufacturing antivenom.
